# The use of genetic programming in the analysis of quantitative gene expression profiles for identification of nodal status in bladder cancer

**DOI:** 10.1186/1471-2407-6-159

**Published:** 2006-06-16

**Authors:** Anirban P Mitra, Arpit A Almal, Ben George, David W Fry, Peter F Lenehan, Vincenzo Pagliarulo, Richard J Cote, Ram H Datar, William P Worzel

**Affiliations:** 1 Department of Pathology, University of Southern California Keck School of Medicine, 2011 Zonal Avenue, HMR 312, Los Angeles CA 90033, USA; 2 Genetics Squared Inc., 210 South 5th Avenue, Suite A, Ann Arbor MI 48104, USA; 3 Department of Internal Medicine, Gundersen Lutheran Medical Center, 1900 South Avenue, La Crosse WI 54601, USA; 4 Dipartimento Emergenza e Trapianti d'Organo, Sezione di Urologia, Università di Bari, Piazza G. Cesare 11, Bari 70124, Italy

## Abstract

**Background:**

Previous studies on bladder cancer have shown nodal involvement to be an independent indicator of prognosis and survival. This study aimed at developing an objective method for detection of nodal metastasis from molecular profiles of primary urothelial carcinoma tissues.

**Methods:**

The study included primary bladder tumor tissues from 60 patients across different stages and 5 control tissues of normal urothelium. The entire cohort was divided into training and validation sets comprised of node positive and node negative subjects. Quantitative expression profiling was performed for a panel of 70 genes using standardized competitive RT-PCR and the expression values of the training set samples were run through an iterative machine learning process called genetic programming that employed an N-fold cross validation technique to generate classifier rules of limited complexity. These were then used in a voting algorithm to classify the validation set samples into those associated with or without nodal metastasis.

**Results:**

The generated classifier rules using 70 genes demonstrated 81% accuracy on the validation set when compared to the pathological nodal status. The rules showed a strong predilection for *ICAM1*, *MAP2K6 *and *KDR *resulting in gene expression motifs that cumulatively suggested a pattern *ICAM1*>*MAP2K6*>*KDR *for node positive cases. Additionally, the motifs showed *CDK8 *to be lower relative to *ICAM1*, and *ANXA5 *to be relatively high by itself in node positive tumors. Rules generated using only *ICAM1*, *MAP2K6 *and *KDR *were comparably robust, with a single representative rule producing an accuracy of 90% when used by itself on the validation set, suggesting a crucial role for these genes in nodal metastasis.

**Conclusion:**

Our study demonstrates the use of standardized quantitative gene expression values from primary bladder tumor tissues as inputs in a genetic programming system to generate classifier rules for determining the nodal status. Our method also suggests the involvement of *ICAM1*, *MAP2K6*, *KDR*, *CDK8 *and *ANXA5 *in unique mathematical combinations in the progression towards nodal positivity. Further studies are needed to identify more class-specific signatures and confirm the role of these genes in the evolution of nodal metastasis in bladder cancer.

## Background

Cancer of the urinary bladder is the seventh most common cancer worldwide (3.2% of all cancers), with an estimated annual incidence of 330,000 new cases and to which 179,000 deaths are attributed each year [1,2]. In the USA, where more than 63,000 new cases of bladder cancer were expected in 2005, urothelial carcinoma (UC) is the most common histology (90%), followed by squamous cell carcinoma (6–8%), adenocarcinoma (2%), and a variety of other rare tumor types [3]. The standard TNM clinical stage classification system for bladder cancer recommended by the American Joint Committee on Cancer takes into account the depth of invasion of the bladder wall by the primary tumor (T), the presence and size of metastatic regional lymph nodes (N), and the presence or absence of distant metastases (M) [4]. Nodal involvement is considered to be an independent risk factor for recurrence and survival after cystectomy for organ-confined bladder cancer [5]. Consequently, extensive bilateral pelvic lymphadenectomy is now considered an integral part of the surgery, having been shown to significantly improve the prognosis of patients with muscularis propria-invasive bladder cancer [6,7]. Non-muscularis propria-invasive tumors (TNM Stages 0a, 0is, and I), confined to the bladder mucosa or subepithelial connective tissue (pTa, pTis, and pT1) without regional (N0) or distant (M0) metastases, are generally treated by transurethral resection of the tumor with fulguration, intravesical chemotherapy, and radiotherapy. Although cures are possible, up to 80% of these presumed "localized" tumors will eventually recur following initial resection, with up to 25% progressing to muscularis propria-invasive disease [8]. The confirmation of the existing true nodal status in a patient with bladder cancer thus assumes primary importance, along with the need to determine if the tumor has the molecular potential to metastasize to the lymph nodes later, provided undiagnosed micrometastasis has not occurred already.

Molecular changes in bladder cancer have been shown to precede morphologic changes that can be identified visually [9]. Further, some tumors have specific molecular patterns that predispose them to be more morphologically aggressive, with a greater propensity to metastasize and recur, regardless of their clinical stage at diagnosis [10]. Hence, morphologic changes need to be complemented with molecular correlates for an accurate prediction of bladder tumor progression.

**Figure 1:**
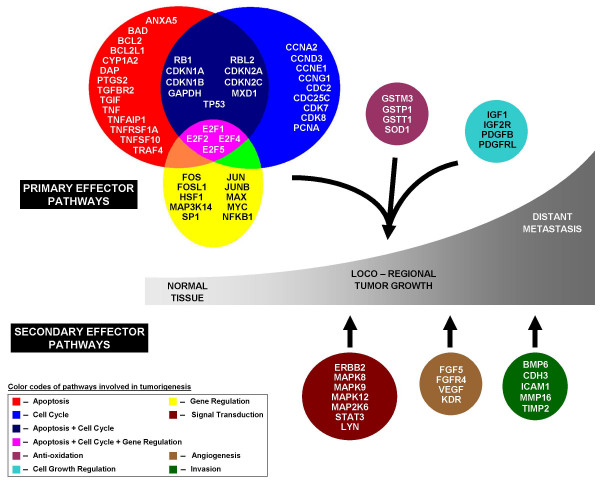
**Marker panel employed for standardized competitive RT-PCR analysis. **A total of 70 genes involved in eight broad pathways commonly deregulated in cancer were chosen for this study. The primary effector pathways of tumorigenesis encompass apoptosis, cell cycle, gene regulation, cell growth regulation and anti-oxidation, and are comprised of 57 genes. There is a significant overlap of markers among the first three pathways. The secondary effector pathways include signal transduction, angiogenesis and invasion, and are comprised of 13 genes. All the listed genes exert stimulatory, inhibitory and/or regulatory effects on their respective pathway(s).

The goal of this study was to create an objective and accurate tool for the identification of nodal status from primary tumor tissue. Since bladder cancer has a multi-factorial etiology with a complex pathogenesis encompassing various pathways that involve more than a simple two directional (up/down) regulation of a few genes, we felt that it was necessary to investigate a comprehensive panel of genes to define this complex disease. Utilizing bladder tissue biopsies from 60 primary UC subjects and five normal controls, our study involved the analysis of a set of 70 candidate markers involved in crucial pathways that have been shown to be deregulated in cancer, including those of cell cycle regulation, apoptosis, angiogenesis, invasion and metastasis, and anti-oxidation [Figure 1, Additional file 1] [11,12]. Since scaling the gene expression levels to represent fold changes relative to a base value could have biased the significance of these gene changes, there was a concern that representing the data in this way might obscure any correlation with the altered gene's function. We, therefore, adopted a standardized competitive reverse transcriptase – polymerase chain reaction (StaRT-PCR™) approach to quantitatively measure gene expression values in relation to a million molecules of a housekeeping gene like β-actin [11]. This gave us an expression profile and molecular signature for each tissue sample with the lowest inter-sample and intra-sample variability.

Using a machine learning technique called genetic programming (GP) [13], the gene expression values were then used to classify the primary tumor tissue samples into those associated with nodal involvement (node positive, NP) and those from subjects known to have no nodal involvement (node negative, NN). GP uses the available data to produce a set of classifiers ("rules") that are optimized in an iterative fashion through successive retention of the better performing rules. One of the key characteristics of GP is its ability to automatically select variables and operators and assemble them into appropriate structures that form predictive functions for classifying the samples, often discovering unusual and unexpected combinations of input variables. In this study, the samples were divided into training and validation sets, and GP was used in a supervised learning mode on the training set to develop a discriminant classifier solution which then used the validation set to test the generality of the solution produced. We, herein, report that by employing GP to analyze quantitative gene expression profiles of primary tumor tissue, one can accurately determine the nodal status of bladder cancer in the same patient, thereby enhancing the ability to correctly assess the extent of disease.

## Methods

### Patient population and distribution

The study cohort was comprised of 60 UC subjects and five normal controls (n = 65). UC tissue was obtained from 50 subjects who underwent radical cystectomy for UC of the bladder at the University of Southern California/Norris Comprehensive Cancer Center from 1997 to 2001 and from 10 subjects who underwent treatment for pTa and pT1 bladder cancer at the University of California, San Francisco. Nodal staging was determined by histopathological examination in the former group and by imaging in the latter. Controls consisted of normal urothelium from the bladder neck of five subjects who underwent radical prostatectomy for prostatic adenocarcinoma localized to the prostate with no bladder involvement at the Norris Comprehensive Cancer Center. None of these subjects had any history of bladder cancer. The 60 tumors had the following stage distribution: pTa (n = 10), pT1 (n = 13), pT2 (n = 8), pT3 (n = 22) and pT4 (n = 7); 21 of these 60 subjects (35%) were NP, although none of the subjects had distant metastatic disease. Subjects with pure adenocarcinoma, squamous cell carcinoma, or small cell carcinoma were not included in the analysis. Nodal status of the subjects was determined during initial diagnosis and after pelvic lymphadenectomy during radical cystectomy for the invasive UC cases. Pathological stage was determined according to the tumor-node-metastasis (TNM) system [4]. Primary tumor samples from the cystectomy specimens were preserved as archival paraffin-embedded tissue blocks and were available in all cases. Informed consent was obtained from all subjects. The gene expression profiling studies were approved by the University of Southern California and the University of California, San Francisco Institutional Review Boards.

The total study population was divided into training and validation sets. The former was comprised of 11 NP subjects and 23 NN subjects, while the latter consisted of 10 NP subjects and 21 NN subjects. The 44 NN subjects included the 5 normal controls which were classified as NN for the purpose of analysis in this study. The distribution of the subjects across both sets and nodal classes was made to maintain an approximately equal proportion across all tumor staging strata [Table 1].

### RNA extraction and cDNA synthesis

RNA was extracted using the TRIzol^® ^method (Invitrogen, Carlsbad, CA, USA). Formalin-fixed paraffin embedded tissue sections were lysed with a syringe in TRIzol^®^. 400 μL of chloroform was then added followed by centrifugation to separate the RNA containing aqueous phase. This was followed by addition of linear acrylamide (Ambion, Austin, TX, USA) that served as a carrier and 1 mL of isopropanol to precipitate the RNA followed by incubation at -80°C for two hours. The tubes were then thawed and centrifuged at 4°C and the supernatant was removed. The RNA pellet was washed with cold 70% ethanol followed by centrifugation and removal of the supernatant. The RNA pellet was dried and resuspended in DEPC-treated water. DNase treatment was then performed using DNA-*free*™ (Ambion, Austin, TX, USA) following the manufacturer's instructions. cDNA was prepared as described previously [14].

### StaRT-PCR™, image analysis and quantitation

Quantitative gene expression profiling was done using StaRT-PCR™ analysis as described previously [14]. The internal standard competitive template (CT) mixtures (A-F) over six logs of concentration were obtained from Gene Express, Inc. (Toledo, OH, USA). While each of the six mixtures (A-F) contained internal standard CTs for 381 target genes in addition to 600,000 β-actin CT molecules/μL, our study targeted a list of 70 transcripts [Figure 1, Additional file 1]. For each sample, StaRT-PCR™ analysis was performed using five different CT mixes (B-F). Thus each sample underwent five separate PCR analyses; each separate reaction containing the ready-to-use master mixture, cDNA sufficient for expression measurements of the 71 transcripts (including β-actin), primers for the 71 transcripts and one of the five CT mixes (B-F). Following PCR, the amplification products were electrophoresed, and image analysis and quantitation of band fluorescence intensities were done as described previously [14].

### Genetic program analysis, voting algorithm and gene usage frequency

GP was used in a supervised learning mode on the training set to develop classifier programs. For this, a "genetic pool" of candidate classification programs was created from which future programs were created through selection and re-combination. The programs were initially created by randomly choosing inputs and arithmetic and Boolean operators that work with the type of inputs selected. A small subgroup of programs was then selected from the main population to create a "mating pool" of programs. Each program was evaluated on input data and the output was a prediction of the nodal status associated with these inputs. The accuracy of a program in correctly classifying the samples according to pre-specified labels was used to calculate a fitness measure for the program. Fitness was determined by calculating the area under the curve (AUC) for the receiver operating characteristic (ROC) of a program generated by the GP system, and evolution was driven to maximize the AUC so as to yield rules with high sensitivity and specificity. The complexity of the rules generated was also restricted to prevent overfitting. This was done by the strict use of mathematical operators (e.g., +, -, x, /, exp), logical operators (e.g., 'and', 'not', 'or') and comparison operators (e.g., =, >, <, ≥, ≤), and gene usage was restricted to no more than seven genes in a solution. 'exp' is an exponent function where exp(N) is equivalent to e^N^. While this may seem odd, it expresses the exponential quality of response relationships between genes, particularly within a pathway. The '?' operator was used as a conditional phrase "IF <predicate> THEN <expression1> ELSE <expression2>."

**Figure 2:**
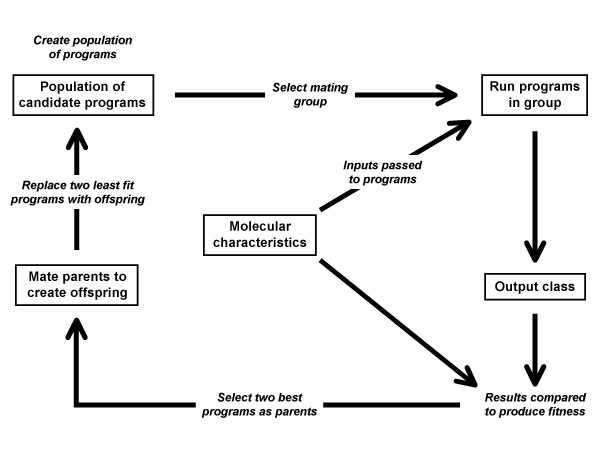
**The genetic programming process. **This iterative technique was employed on the training set samples to generate classifier rules that were tested on the validation set. Randomly chosen components were initially used to create a population of candidate programs from which a small mating pool of candidate programs was generated. Inputs were passed into these programs and the predicted nodal statuses were evaluated for fitness. The two best performing programs were then mated to produce offspring that replaced the two least fit programs. This process was repeated over many generations to create better programs.

The two programs in the mating pool with the highest fitness values were then chosen for selective combination (i.e., mating) to produce offspring. The offspring programs then replaced the least fit programs in the main population, potentially containing superior traits taken from each of their parents. This was repeated and, over many generations, progressively better programs were created [Figure 2]. The relatively small data set also necessitated the employment of a cross validation technique to estimate the ability of the classifier to generalize to unseen samples, giving an approximation of its robustness. This was done using the N-Fold cross validation technique wherein the training set was subdivided into eleven "folds" (N = 11), as there were eleven NP cases in the training set. Classifier rules were developed using the samples in 10 folds and each rule was then tested on the eleventh fold of the training set. The process was repeated 10 more times with each fold taking a turn as the test fold. 20 runs of 11 folds each were completed and the set of classifiers that had the best total performance across all the folds was then selected. As GP is a stochastic process and gives more than one solution with the same accuracy, this gave a reasonable sample of the best performing classifier sets. Classifier sets ("meta-rules") were then used in a majority voting scheme to classify the samples in the validation set. Aggregate performance of these meta-rules on the test folds was taken as the predictor of the classification error, and the selected meta-rule was the one with the least test error. The 11-fold cross validation resulted in a meta-rule for each run that was composed of eleven rules, one for each fold. The meta-rule then "voted" for a sample presented to it. If the majority of the rules (i.e., six or more) voted that the sample belonged to the target class (in this case, NP), the meta-rule was designated as predictive of the target class.

Running the expression values of the training set samples 20 times over 11 folds resulted in 220 rules, each of which had five genes on an average. Thus, the postulated frequency of occurrence of each of the 70 genes in 220 rules if all had equal probabilities of being selected was 15.71. The actual frequencies of occurrence of all genes were recorded by identifying the number of instances amongst all the 220 rules, wherein a rule had the gene as one of its constituents. Since the occurrence of each gene in a rule was a binary event, the frequency of each gene being selected followed a binomial distribution enabling the calculation of binomial probabilities and their corresponding p-values. Statistical calculations were performed using SAS (release 9.1).

## Results

### Generation of rules

Quantitative gene expression profiling of the tissue samples for the above mentioned 70 genes was done using StaRT-PCR™ and the expression values for the samples grouped under the training set were run 20 times through the GP system for 100 generations over 11 folds to yield meta-rules. The fitness measure tried to maximize the AUC while overfitting was avoided by using simple mathematical functions and restricting rule sizes as described above. N-fold cross validation was used as the resampling technique to test the overall generality of the classifier. The generated rules were then subject to a majority voting algorithm and the best performing rules were chosen and tested on the validation set against the histopathologically determined nodal status. The accuracy of each meta-rule was assessed by calculating how well it classified the validation set samples based solely on their molecular characteristics. The final meta-rule thus generated is shown in Table 2 which correctly identified 6 out of 10 NP samples and 19 out of 21 NN samples in the validation set, resulting in a positive predictive value of 75% and negative predictive value of 83% [Table 3]. All of the normal cases, classified as NN, were correctly identified as being node negative, yielding an overall sensitivity of 60% and a specificity of 90%.

### Gene usage

**Figure 3:**
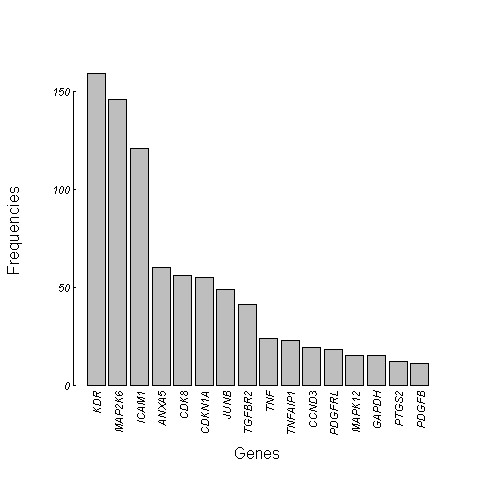
**Histogram of Gene Usage Frequencies. **Examination of the gene usage frequencies among the best of 220 rules drawn from 20 runs of 11 folds showed a strong preference for the *KDR*, *MAP2K6 *and *ICAM1 *genes which were also components of some of the major gene expression motifs. Rules created using only the top three genes showed a comparatively better performance, indicating their importance in the genesis of nodal metastasis.

Cross validation consistency is a concept that implies that the presence of a fundamental phenomenon implicit in the data will be reflected by a similarity between the results for different folds [15]. The frequency of gene usage in the best rules across many runs with different fold compositions of the data thus allowed us the possibility of identifying the most important genes as well as gene-gene interactions used in the classifiers for target discrimination. Although the stochastic nature of GP combined with the fact that the fold composition is partly determined by random selection may lead one to assume that it might be possible to craft a rule that could classify any limited number of samples given a random selection of genes, this proves not to be the case. We show that repeatedly running the system and tabulating the use of genes across all folds in all runs reveals a strong preference for certain genes. The genes *KDR*, *MAP2K6 *and *ICAM1 *(encoding the kinase insert domain receptor, mitogen-activated protein kinase kinase 6, and intercellular adhesion molecule 1, respectively) showed a strong predilection to be used among the 220 rules drawn from 20 runs of 11 folds [Figure 3]. Since the presence or absence of a gene in a rule is a binary event, the frequency of each gene being selected follows a binomial distribution. The binomial probabilities of the above genes were 9.69E-130, 1.13E-110, and 4.10E-78, respectively, with one-sided p-values of <0.00001 against the null hypothesis of each of the 70 genes having an equal probability of being selected [Table 4]. The p-values for the next five genes were also low enough to indicate significant selectivity towards them. Examination of the rules presented in Table 2 show the prevalence of the top three genes where each rule on this list uses at least one of these genes and four of the rules use all three genes, though in different combinations.

### Gene expression motifs

In addition to raw gene frequency information, the rules developed were examined for recurring mathematical combinations that may be called "motifs". These not only showed the tendency to utilize certain genes more than others but also demonstrated the relationship between these genes. From this, it may be possible to identify pathways that are associated with the signature of a certain target class, which in this study was the class of node positive subjects.

Examination of the rules revealed a consistent relationship between the expression levels of the three most frequently utilized genes, i.e., *KDR*, *MAP2K6 *and *ICAM1*. That is, the more highly expressed *MAP2K6 *and *ICAM1 *were when compared to *KDR*, the more likely it was that a sample would be NP. These, however, are relative comparisons. In other words, *KDR *is lower when compared to *MAP2K6 *and *ICAM1*, but a simple assumption of low *KDR *expression in general cannot be construed as a marker of nodal positivity (see Additional file 2). An example motif that shows this relationship is the expression '*MAP2K6*/*KDR*'. The rules developed all take the form: "IF [mathematical expression] ≥ [threshold] THEN NP". In this case, a lower *KDR *expression value translates to a higher ratio with an increasing likelihood of the sample being classified as NP. Similarly, the higher *MAP2K6 *is when compared to *KDR*, the more likely it is that this expression will be greater than the constant value. This motif occurs in rules 2, 4, 7 and 11 in Table 2. A similar motif that was often observed was '*MAP2K6 *- *KDR*', as in rules 5 and 1 [Table 2]. (Note that in Rule 1, the relationship is reversed because it appears in the denominator of the ratio.) Again, *KDR *would tend to be mathematically lower than *MAP2K6*.

The motif '*MAP2K6*/*ICAM1*' and its related motif '*ICAM1 *- *MAP2K6*' appear quite frequently, with the former used in a reductive way (i.e., reducing the value that is compared to the constant value) and the latter in an additive way (i.e., increasing the value that is compared to the constant value). This suggests that in NP cases, *ICAM1*>*MAP2K6*. Since we had previously identified the relationship *MAP2K6*>*KDR *from the use of the *MAP2K6 *and *KDR *motifs described above, we can infer that in NP cases *ICAM1*>*MAP2K6*>*KDR*. This might be called "gene transitivity" in the sense that since *MAP2K6 *is greater than *KDR *but less than *ICAM1*, we can infer that *ICAM1 *is in general greater than *KDR *in NP cases. This relationship may be seen in rules 2 and 5, respectively [Table 2].

Another motif observed was '*ICAM1 *- *CDK8*' or the similar motif '*ICAM1*/*CDK8*', which were featured in rules 3, 4 and 6 [Table 2]. The prevalence of these motifs shows that a high *ICAM1 *value relative to *CDK8 *(which encodes cyclin-dependent kinase 8) is likely in NP samples. Rule 10 shows the motif '*CDK8*/*ICAM1*', but as this expression is being subtracted from the rest of the expression, it also leads to the same conclusion of *ICAM1 *levels tending to be higher relative to *CDK8*. There was no obvious motif that linked *CDK8*'s relationship to either *MAP2K6 *or *KDR*.

Finally, though not technically a motif, the fourth most frequently used gene, *ANXA5*, that encodes for annexin A5, appeared in a variety of combinations in rules 4, 9 and 10 [Table 2] and a relatively high expression value of the gene was generally associated with NP cases.

### Identification of a single rule

While the voting algorithm seems to work reliably, there is a natural desire to identify the "best" rule for classifying samples. While this may be contrary to the population-based process used by GP, the gene usage frequency results indicate that the top three genes are used significantly more often than the others. This suggests that these genes play the most important part in distinguishing NP samples from NN samples. One hypothesis could be that these genes are carrying the bulk of the value in the rules presented. To investigate this idea further, rules were created using only these three genes viz., *KDR*, *MAP2K6 *and *ICAM1*, as inputs. The entire GP process was repeated in order to clearly identify the relationship of these three genes and to test the robustness of the rules developed from this subset of genes. The final meta-rule obtained after 11-fold cross validation comprised of eleven rules (see Additional file 3).

The performance of these rules on the validation set was found to be comparable to the previous results generated using the panel of 70 genes, demonstrating equal levels of accuracy and comparable sensitivity and specificity metrics [Table 5], which tend to support the hypothesis that rules created with a reduced number of genes that are believed to be the key modulators of the target process generate equally robust classifiers. Of note is the fact that the first rule from the generated meta-rule (see Additional file 3) when singularly analyzed retrospectively on the validation set, produced a markedly better result with 70% sensitivity and 100% specificity. The positive predictive value of this rule was 100% and the negative predictive value was 88%, though there was nothing that uniquely stood out about this rule in comparison with the others (see Additional file 4). However, the observation that most of the rules show similarity in constitution with respect to gene usage further bolsters the hypothesis that these genes are critical in the development of nodal metastasis and interact with each other in distinct effector pathways.

## Discussion

Recent studies suggest that the significant relapse rates for bladder tumors that do or do not invade the muscularis propria may be related to the presence of micrometastases in pelvic lymph nodes that are undetectable using conventional computed tomography, magnetic resonance imaging, positron emission tomography and routine histopathologic examination [16,17]. Hence, consideration for early cystectomy with pelvic lymphadenectomy is now being advocated even for "localized" bladder cancers that have not invaded the muscularis propria [18]. A more accurate definition of the nodal status upon initial diagnosis and during follow-up of bladder cancer will go a long way in minimizing the significant understaging and overstaging that appears to currently exist and thereby better equipping the clinician with the tools needed to determine the optimal treatment and follow-up strategies for a particular patient.

Over the past decade, efforts have begun to identify molecular markers that can predict the propensity of bladder tumors to metastasize to the lymph nodes. While single molecular markers with significant correlations have been identified, the predictive and prognostic potential offered by them is still not optimal. The current situation warrants the need to generate a panel of markers representing those crucial pathways deregulated in bladder cancer which can assist in the prediction of nodal metastasis. The present study evaluates a panel of 70 transcripts that are known to be altered in cancers. The expression levels of these genes were determined using StaRT-PCR™ and the data was subjected to GP analysis, which identifies optimal rules using those genes that it selects as the most significant determinants of the target clinical outcome (in this case, nodal metastasis). StaRT-PCR™ has the ability to measure the stoichiometric relationship between the abundance of multiple transcripts within the same sample [11] and can allow for comparison of data generated independently in different experiments and different laboratories [19].

### Considerations involved in construction of the study cohort

The total study cohort of 65 subjects was divided into training and validation sets, and an approximately equal distribution was attempted between them for each nodal class within a tumor stage in an effort to eliminate bias [Table 1]. Besides the five normal samples, the rest of the cohort (n = 60) thus has the following distribution: 20 NN cases and 3 NP cases in the non-muscularis propria-invasive category (pTa and pT1); and 19 NN cases and 18 NP cases in the muscularis propria-invasive category (pT2-4). The cohort thus exhibited an equal proportion of NN and NP cases in the muscularis propria-invasive category, but an unequal proportion of the same in the non-muscularis propria-invasive category. These proportions are reflected in the subject distributions in the training and validation sets, and may prompt one to surmise that the gene selection process was biased as it recognized tumor stage-specific features rather than those for nodal status. However, given the approximately equitable distribution of NN cases between the non-muscularis propria-invasive and invasive groups, one can conclude that the features identified by GP corresponded to the absence of nodal metastasis rather than detrusor muscle invasion or tumor stage. The inequitable distribution of NP cases might lead one to believe that the features identified by GP may correspond more to the presence of detrusor muscle invasion (as the number of muscularis propria-invasive cases are higher) rather than the presence of nodal metastasis. This would, however, mean that GP would not be able to distinguish between NN and NP pTa and pT1 cases, as all these cases would demonstrate common features of lack of muscle invasion. However, the stage-wise break-up of the results show that each time GP was run, it able to distinctly identify between NN and NP pTa and pT1 cases with 100% accuracy (see Additional file 5). Indeed, the paucity of NP non-muscularis propria-invasive cases is common in clinical settings as only a small minority of non-muscularis propria-invasive cases metastasize to the lymph nodes at the time of diagnosis [6]. Alternatively, NP cases are generally considered to be more aggressive and thus usually present with a greater degree of tumor invasion. The study cohort was so constructed to reflect this clinical scenario, albeit using a small number of subjects.

The normal subjects were clubbed with the NN cases to confirm that the technique could recognize normal samples as NN as well. While the genetic makeup of normal urothelium may be entirely different from NN UCs, the common theme was the absence of nodal metastasis (and thus, an absence of the genetic features contributing to the same), rather than the presence or absence of carcinoma afflicting the urothelium. Amalgamation of the two groups was thus crucial to create a binary classification system that distinguished the presence or absence of nodal metastasis.

### Considerations involved during generation of rules

Transcript levels were used to generate classifier rules that were produced after evolving over 100 generations until they reached an acceptable level of accuracy. It is necessary to provide a suitable fitness measure, as fitness is the main driver of the evolutionary process and thus determines the quality of solutions achieved. One possible measure of fitness is a calculation of sensitivity and specificity in a rule's ability to predict the NP subjects. The problem in selecting a single measure of accuracy out of sensitivity and specificity is that they are both inherently complementary, wherein increasing one is often associated with decreasing the other. The overall objective of simultaneously maximizing both parameters was built into the ROC evaluation of the test [20], and the search for the most informative test sought to maximize the AUC [21]. The AUC gives a direct indication of how well the samples are being separated into different classes and is thus a more robust fitness measure than any other mathematical combination of sensitivity and specificity because there is no concept of boundary or threshold that can induce discontinuities into the system leading to strange behavior around them.

A major challenge in any machine learning system is to prevent overfitting. This occurs when the function is biased strongly towards training examples and generalizes poorly to new (unseen) examples. Typically, model overfitting occurs when there are too few samples relative to the complexity of the problem. Most clinical problems must deal with this issue. In our study, there were only 34 samples in the training set from which to learn, with 70 variables per sample. This could potentially have led to solutions that could have been overly biased or overfitted to the training data. This study tried to alleviate the overfitting problems by restricting the complexity of the result and by the use of resampling techniques. By restricting the complexity of the result, the system was forced to pick out the most salient features in the data set which were likely to be the most general solutions [22]. This was achieved by the use of simple mathematical, logical and comparison operators, and by limiting the size and complexity of the programs produced based on the minimum description length principle of risk minimization wherein the least complex solution is called the most robust [23]. The number of genes used in any solution was also restricted to no more than seven, which puts a constraint on the degrees of freedom in the expression that is loosely related to the VC dimension, a measure of the complexity of a classification algorithm [24].

For selecting a robust classifier, it is imperative to know the generalization rate of the classifier, especially in the case of small data sets where overfitting can be relatively frequent. Cross validation is a resampling technique that can help predict the generality of the solution in classifier problems [25]. In cross validation, the training set is subdivided into N subsets or "folds" and then each of the N - 1 sets are used to learn from and the N^th ^fold is used to test the resulting rules. The folds are randomly assembled from the whole data set, maintaining the same proportion of true positive cases and true negative cases such that each fold will have the same representation of samples as the whole set. To avoid selecting a particularly favorable test subset, the system is then run again from scratch with another fold as the test set. This is repeated until all N subsets have been used as the test set. The goal is to adjust the system so that the results for all training-test set combinations (folds) are roughly the same.

While N-fold cross validation is a simple and effective technique to evaluate how well the classifier generalizes to unseen data, the number of folds to use in order to best assess the general performance of the system is an open question. Many machine learning techniques use a leave-one-out cross validation (LOOCV) [26] approach as it has the virtue of maximizing the use of samples by allowing the investigator to view the overall performance of the system across many folds, suggesting a "normal" behavior for the rules generated. LOOCV is approximately unbiased for true prediction error, but can have high variance because all the "training sets" are similar to each other. This study used a variation of cross validation inspired from the N-fold cross validation scheme that selects an optimum number of folds that can strike a reasonable balance between bias and variance. Instead of having 34 folds, which would correspond to the total number of samples in the training set, 11 folds (i.e., the number of NP samples in the training set) were used that lead to a reduction in the variance of the solution. The best performing classifiers across all folds were ultimately selected and applied to a majority voting scheme to generate the best meta-rule. The majority voting scheme increases the performance and consistency of the classifiers. It has been shown that for a binary classification scheme, the performance of the aggregate classifier actually increases if the individual rules are more than 50% accurate [27,28]. Resilience can be significantly improved with this approach as estimation errors are reduced.

### Clinical relevance of the frequently used genes

The panel of 70 genes was chosen based on an extensive review of previous studies that have implicated potential roles for various genes in the progression of cancer in general and UC in particular. Although many of the candidate markers that were selected for this analysis have been implicated in one or more of the pathways involved in bladder tumorigenesis, it is plausible that some genes may play a relatively more significant role in the development of nodal metastasis and the determination of prognosis if the disease is detected early. The GP analysis in this study clearly shows an unequivocal preference to use *ICAM1*, *MAP2K6*, *KDR*, *CDK8 *and *ANXA5 *in specific relationships to define NP UC specimens. The association of metastatic disease with the expression levels of these genes and their corresponding proteins is not unreasonable considering their function and involvement in tumor biology.

ICAM1 is a cell surface glycoprotein in the immunoglobulin superfamily and is expressed at a low basal level in fibroblasts, leukocytes, keratinocytes, endothelial and epithelial cells but is upregulated in response to a variety of inflammatory mediators [29] Several reports indicate that the expression levels of ICAM1 correlate with metastatic potential, migration, and infiltration ability. Immunohistochemical studies on 57 patients with bladder carcinomas revealed that the expression of ICAM1 was closely associated with an infiltrative histological phenotype [30] and serum ICAM1 levels have been related to tumor presence, grade and size in patients with bladder cancer [31]. Furthermore, fibrinogen, which may be a determinant of metastatic potential [32], mediates bladder cancer cell migration through an ICAM1-dependent pathway [30]. More recently, it was shown that ICAM1 downregulation at the mRNA and protein levels led to a strong suppression of human breast cancer cell invasion through a matrigel matrix and that the level of ICAM1 protein expression on the cell surface positively correlated with the metastatic potential of five human breast cancer cell lines [33].

Ligation of ICAM1 induces activation of MAP2K6, which in turn activates p38 [34-36]. This pathway has been shown to be closely associated with an invasive phenotype for bladder tumors [37] and p38 phosphorylation in breast cancer patients has been associated with a poor prognosis in node-positive tumors [38]. Other studies have shown a direct effect of MAP2K6 activity on metastatic potential. MAP2K6 transfection into normal MCF10A breast epithelial cells resulted in an invasive and migratory phenotype accompanied by upregulation of certain matrix metalloproteinases [39,40]. Activation of this pathway also induced in vitro invasion of normal NIH3T3 fibroblasts [41].

KDR or vascular endothelial growth factor receptor-2 (VEGFR2/Flk-1) is a high-affinity plasma membrane receptor for the ligands VEGF-A and -E. This protein is expressed on endothelial cells in the vasculature and mediates most of the endothelial growth and survival signals through these ligands [42]. Expression of KDR has also been demonstrated in tumors of epithelial origin and the best rules in this study imply that the expression level of *KDR *is consistently lower in relation to *ICAM1 *and *MAP2K6 *when there is nodal involvement in bladder cancer. Although a precise reason for why this relationship should exist is unknown, some studies have established a more aggressive phenotype in cancers that have lower expression of KDR. For example, in patients with UC, high expression of KDR was associated with increased survival times, whereas those with lower expression values had a worse prognosis [43]. Likewise, high-grade prostate carcinomas showed much less KDR expression than low or moderate grade tumors [44]. However, it must be kept in mind that in the present study, it is not the absolute expression of *KDR *that contributes to the correlation with nodal involvement in bladder cancer, but its relationship to the values of the other two genes.

The identification of CDK8 as a physiological partner of cyclin C (CycC) is relatively recent [45] and the role of the former with respect to clinical prognosis in UC has not been extensively investigated. The CycC/CDK8 complex is a part of the pol II holoenzyme complex that plays a part in transcription [46], and is also a part of MED/SRB (Mediator/Suppressor of RNA Polymerase B) containing complexes such as TRAP/SMCC (Thyroid hormone-associated protein/SRB/MED cofactor complexes) and NAT (negative regulator of activated transcription) [47-50]. TRAP/SMCC and NAT have been shown to phosphorylate CycH of the general pol II transcription factor, TFIIH complex, via their CDK8 kinase activity and inhibit TFIIH protein kinase activity [51]. The suggestion of a lower *CDK8 *level compared to *ICAM1 *in NP cases through the gene expression motifs in this study may thus be suggestive of a role of increased transcription activity, though more functional studies in this direction are required.

The annexins are a large family of closely related calcium- and membrane-binding proteins [52] that are expressed in most eukaryotic cell types and appear to participate in a variety of cellular functions including vesicle trafficking, cell division, apoptosis, calcium signaling, and growth regulation. Many of these proteins are differentially expressed in malignant tissue and have been shown to be upregulated or downregulated depending on tumor type [53]. Annexin V (and in the case of our study, annexin A5, encoded by the *ANXA5 *gene) has been reported to be especially abundant in platelets where it relocates to the cytoskeleton following stimulus-induced Ca^2+ ^elevation [54,55]. Annexin V is used as a marker for apoptosis [56] and has been shown to influence susceptibility to apoptosis and pro-inflammatory activities of apoptotic cells [57]. Consequently, annexin V expression levels could be affected by the apoptotic potential of a tumor cell population, which has been shown to be greatly influenced by the process of tumor progression and metastasis [58].

Interestingly, previous studies that have attempted to identify prognostic classes of UC using microarrays have limited similarity to the gene panel in this study, with *E2F4*, *PCNA*, *CCNA2 *and *RB1 *being upregulated in high grade pTa tumors, and *ERBB2 *being downregulated in muscularis propria-invasive tumors [59]. However, one must note that such studies generally consider tumor grade and stage as prognostic indicators, and their molecular signatures are usually filtered to reflect the same. On the other hand, our study defined nodal metastasis as a prognosticator and the GP system was thus trained to identify genetic traits that best corresponded to this indicator. Furthermore, the identification of a single rule employing the three most frequently used genes proved to be more robust in terms of predicting the nodal stage in the validation set. While this study suggests a possible pivotal role of the expression of these three genes in determining nodal status, the limitation of a relatively small sample size compared to the number of variables involved warrants similar studies with larger sample sizes to validate these results. One can hypothesize that comparative downregulation of *KDR *(and perhaps, angiogenesis in general) in the primary tumor might impart selection pressure for invasion leading to upregulation of *ICAM1 *that reflects a tumor's potential to establish a metastatic lesion in a draining lymph node. This possible mechanism can partially explain the gene transitivity observed, whereby the gene expression signature of nodal metastatic cases consistently conforms to a fixed motif of *ICAM1*>*MAP2K6*>*KDR*, although a literature search did not reveal any study that investigated these genes in concert in the context of nodal metastasis. The above motif also exemplifies the hypothesis-generating nature of GP, and directed investigations into the role of these genes and their respective pathways in promoting nodal metastasis are required. Further work is necessary to show that this approach is effective in all cases but it tends to support the theory put forth by Daida that GP has two phases: identifying the key inputs, and then finding rules that optimally combine them [60]. By using specific genes shown to be more useful during pilot studies, it allows the GP process to search for the best use of those genes. This can enable the GP system to accurately predict prognosis and help in making therapeutic decisions, thus having a direct impact on the patient and the treating clinician.

### Advantages of genetic programming

As can be seen from the results, GP is distinct from other common machine learning algorithms used in bioinformatics [Table 6]. This technique is gradually gaining popularity for the analysis of medical and biological data, and for the prognostic classification of cancers [61-65].

A unique feature of GP is the final output, which consists of easily readable rules expressed as executable classifier programs that define tangible relationships between the most influential genes. This allows the results to be put into the known biological context of these genes, which can enhance their significance or provide new working hypotheses that could be further tested. Most other classifier algorithms like Support Vector Machines (SVM), Neural Networks and K-Nearest Neighbors (KNN) clustering approaches do not provide human readable results. Hierarchical clustering creates visually intuitive results but the output does not specify an exact relationship amongst the genes. Classification and regression trees (CART) output a binary decision tree that comes closest to GP developed rules in terms of human readability but fail to provide clear insights into gene co-expressions that become more difficult to discern due to the relationships becoming less explicit as the trees grow larger. While CART algorithms are normally felt to be greedy, aiming to locally optimize the decision tree during construction of the solution [66], GP takes on a more global view of the solution space and can thus search a larger space for solution trees that might lead to improved performance.

By identifying those genes that are most dominant in defining outcome, the GP process can usually limit the complexity of the classifiers and generate robust but simple rules containing only a few genes without compromising their predictability. This is potentially useful in a clinical setting where profiling each gene has its own cost. The smaller the gene set needed to make a clinical diagnosis, the cheaper the test and potentially the more acceptable it is to payors.

GP does not assume extensive prior knowledge on the expected form of the solution or any preconceived genetic interactions to set up the analysis. This is especially useful given that genetic relationships are not always well known. Hence, one can judge how it could be difficult to use classification or clustering algorithms where one needs to pre-specify the structure of the expected solution. For example, SVMs involve selecting a kernel for mapping the data to a higher dimensional space, which is non-trivial and often a non-intuitive process that can affect the accuracy of the classifiers.

GP can also select variables automatically without any need to pre-filter or limit them based on what is known about a system. Such filtering is usually done because of the combinatorial problem of working with a large number of inputs; however, such filtering can create an incomplete and biased dataset that may not be representative of many complex biological systems. The "curse of dimensionality" [67] affects all classification algorithms but the problem of dimensionality reduction is more important in classical algorithms like hierarchical, KNN, K-means clustering and Neural Nets which do not scale easily to larger numbers of variables. Feature selection is then an important step before the application of these algorithms and can lead to loss of information that might be critical for the success of the learning algorithm.

As can be seen from the rules generated in our study, most rules express non-linear relationships such as *MAP2K6*/*KDR *or *ICAM1*/*CDK8*. The ability to choose variables from a large list and then combine them in a non-linear, readable way is a powerful feature of this approach as many biological systems often have non-linear relationships between genes or proteins. SVM is a popular algorithm which outputs non-linear classifiers but is limited by the kernel selected. CART algorithms implement non-linearity in a pseudo sense as they split the data and tackle each partition separately, but are not as succinct as the rules produced by GP in capturing the effects and the relationships among gene expressions.

Lastly, GP can incorporate very diverse data sets that contain markedly different types of variables and can also handle missing values in the data. Missing data can be an important problem as even a small amount of missing data can lead to a large loss in performance. This is especially true in systems like Hierarchical or KNN based clustering and SVMs, that necessitate the use of various tools like imputation, replacement of the missing values with a constant, and removal of samples with a large amount of missing data. However, most of these approaches can introduce bias in the system due to the assumptions made about the missing data that could lead to loss of important features. GP alleviates this problem by leveraging the ability of the system to automatically select features. Whenever a rule encounters missing data in a sample during fitness assessment, the sample is labeled as misclassified, thus decreasing the fitness of the rule. Thus, the system is not favorably disposed towards picking up a variable (feature) that is laden with a large percentage of missing values. This approach allows for maximum use of the available data without making any unwarranted assumptions about missing data.

### Limitations of genetic programming

GP is a computationally intense process requiring a large amount of machine time. The estimated machine time increases with increasing complexity of the problem, and increase in the dimensions and number of samples. This can be resolved by using parallel processing and segmenting the problem into parts which can be performed on different processors simultaneously and then synchronizing among them. GP is particularly tractable for parallel computing techniques as there are several natural ways to distribute execution onto different machines [68].

As GP is a stochastic process that depends highly on the initial control parameter settings, it does not guarantee an optimal solution in all runs. It should therefore be run several times with different settings to ensure that the system has not fallen into a local optima.

While GP combines features of global and local search algorithms, the cost is that it often performs neither of these functions as well as more specialized algorithms. The constant introduction of new genetic material through mechanisms of mutation and crossover (mating) will divert the algorithm from finding the best combination of a few highly effective components. For this reason, this study adopted a two-phase strategy where the most important variables were identified from the list of the most frequently chosen variables [Figure 3] and the system was then run again using only those high frequency variables. The first pass allowed the GP system to globally search a large set of possible variable combinations while the second pass let it locally search for the best combination of those variables.

GP may also output several rules that are quite different but perform equally well, thus suggesting the involvement of multiple and often unrelated genes. The selection of a single rule can be difficult, particularly when searching for a general solution to a problem. This led us to adopt the voting algorithm to tackle the problem of rule selection and consistency. It would also seem logical to be relatively sure of the biological functionality of the genes selected unless there is sufficient data to confirm an unusual rule or gene selection. This is not a limitation of GP per se, but rather a limitation of any machine learning algorithm.

## Conclusion

Our study uses UC as a clinical model in devising a strategy to combine the medium-throughput quantitative StaRT-PCR™ technique with supervised GP methods to determine the nodal status of clinically diagnosed tumors based on their molecular profiles. We demonstrate that StaRT-PCR™ can provide a relatively standardized output of quantitative gene expression relative to a housekeeping gene like β-actin and can be used as an input in a GP system to generate a classifier for nodal status with a reasonable degree of accuracy. Moreover, the output has also suggested a key role for specific genes involved in the target process that may lead to future studies to clarify their precise biological role and identify new targets for therapeutic intervention. Of particular interest are the gene expression motifs which have identified novel relationships between specific genes and pathways. The key genes identified by this technique from our data set also suggest that class-specific signatures using a small number of genes can characterize tumors as NP or NN, and more importantly, provide an early indication of their progression towards NP status based on molecular traits.

Our group is currently addressing several open questions in GP including an approach for multi-class problems, automated methods for selecting key transcripts and automated identification of significant motifs. Further studies will be aimed at correlating molecular markers and motifs with clinical outcome in an effort to employ markers as reliable, reproducible and objective indicators of prognosis. The enhanced value of incorporating molecular markers into the existing clinical staging of bladder cancer has already been proposed as a prudent alternative [69]. GP will then be ultimately useful in the identification of new avenues of molecular investigations, critical components and signatures of prognosis, and therapeutically feasible targets.

## Abbreviations

UC → urothelial carcinoma

StaRT-PCR™ → standardized competitive reverse transcriptase – polymerase chain reaction

GP → genetic programming

NP → node positive

NN → node negative

CT → competitive template

AUC → area under curve

ROC → receiver operating characteristic

KDR → kinase insert domain receptor

MAP2K6 → mitogen-activated protein kinase kinase 6

ICAM1 → intercellular adhesion molecule 1

CDK8 → cyclin-dependent kinase 8

*ANXA5 *→ annexin A5 (gene)

LOOCV → leave-one-out cross validation

VEGF → vascular endothelial growth factor

CycC → cyclin C

MED/SRB → mediator/suppressor of RNA polymerase B

TRAP/SMCC → thyroid hormone-associated protein/SRB/MED cofactor complexes

NAT → negative regulator of activated transcription

SVM → support vector machines

KNN → K-Nearest Neighbors

CART → classification and regression trees

## Competing interests

Authors AAA, DWF, PFL and WPW are employees of Genetics Squared Inc. (Ann Arbor, MI, USA). RJC is a member of the scientific advisory board for Genetics Squared Inc., which is developing the commercial uses of the genetic programming system used in this study. Other authors declare no competing interests.

## Authors' contributions

APM was responsible for organization of the study cohorts, genetic programming outcome analysis, investigation of molecular biology and clinico-pathological correlations and co-drafting the manuscript. AAA was responsible for genetic programming analysis of the data and co-drafting the manuscript. BG and VP carried out the StaRT-PCR™ experiments. DWF researched and co-authored the section on the clinical relevance of the frequently used genes and critically revised the manuscript. PFL provided background clinical information on bladder cancer, and the rationale and potential significance of the study from a clinical oncology perspective, and critically revised the manuscript. RJC and RHD were responsible for designing the StaRT-PCR™ experiments, and participated in the design and coordination of the study. WPW directed the computational part of this study, drafted portions of the computing sections of the manuscript and participated in overall editing. All authors read and approved the final manuscript.

## Pre-publication history

The pre-publication history for this paper can be accessed here:



## Supplementary Material

Additional file 1 A comprehensive list of the genes investigated using StaRT-PCR™ including their GenBank accession, GeneID and UniGene cluster numbers. Click here for file

Additional file 2 The box plots for the gene expression values for *KDR*, *MAP2K6 *and *ICAM1 *that are differentially expressed in the primary tumor tissues of the node positive and the node negative classes. Click here for file

Additional file 3 The classifier rules that form the meta-rule generated for node positive patients using the three most frequently used genes, viz. *KDR*, *MAP2K6 *and *ICAM1*. Click here for file

Additional file 4 The performance metrics of the first classifier rule from the meta-rule generated for node positive patients using the three most frequently used genes, viz. *KDR*, *MAP2K6 *and *ICAM1*, on the validation set. Click here for file

Additional file 5 The stage-wise and case-wise performance of the meta-rules generated using 70 genes and the three most frequently used genes, viz. *KDR*, *MAP2K6 *and *ICAM1*, on the validation set. This file also shows the stage- and case-wise performance of the single rule "(*MAP2K6*/*KDR*) × (1.0 - (*MAP2K6*/*ICAM1*)) > .71" selected from the meta-rule generated using the three most frequently used genes on the validation set. Click here for file

## Figures and Tables

**Table 1:** Distribution of the study population on the basis of nodal positivity and tumor stage.

**Tumor Stage**	**Training Set**			**Validation Set**		
	
	**Node positive**	**Node negative**	**Total**	**Node positive**	**Node negative**	**Total**
Normal controls		3	3		2	2
pTa	0	3	3	0	7	7
pT1	2	6	8	1	4	5
pT2	0	4	4	0	4	4
pT3	7	5	12	7	3	10
pT4	2	2	4	2	1	3

**Grand total**	11	23	**34**	10	21	**31**

The total cohort of 65 subjects included five normal controls that were classified as node negative. An approximately equal distribution of the subjects was attempted between both sets in all tumor stages and nodal classes to eliminate bias. Tumor and nodal stages was determined according to the American Joint Committee on Cancer recommended TNM system for urinary bladder cancer (2002).

**Table 2:** Final meta-rule for node positive patients generated from the set of 70 genes.

**Rule number**	**Classifier Rule**
1	exp(exp(*HSF1*)) - exp(*MXD1*)/(*KDR *- *MAP2K6*) > 2.718
2	(*MAP2K6*/*KDR*) × (exp(*TGIF*) - *MAP2K6*/*ICAM1*) > .709
3	(*ICAM1 *- *CDK8*)/(exp(*JUNB*) × (*JUNB *- exp(*TGFBR2*))) > 1.32
4	*ANXA5 *× *MAP2K6*/(*KDR *× (*ICAM1 *- *CDK8*)) > 1.701
5	(*ICAM1 *- *MAP2K6*) × exp(*MAP2K6 *- *KDR*) > 3653.813
6	(*ICAM1 *- *CDK8*) × *TP53*/(exp(*TGFBR2*) × *PTGS2*) > 21941.453
7	(*CCND3*/*MAP2K6*) × (exp(*BMP6*) - (*KDR*/*MAP2K6*)) > .201
8	*MAP2K6*/(*CDKN1A *× exp(*MAPK12*) × (*CDC25C *- *KDR*)) > 7.703
9	(*ANXA5 *- exp(*PDGFRL*))/(*CDKN1A *× (*KDR *- exp(*TGFBR2*))) > .044
10	*ANXA5*/(*CDKN1A *× (exp(*PTGS2*) - (*CDK8*/*ICAM1*))) > 79.002
11	*MAP2K6*/(*KDR *× (*ICAM1 *- (*TNFAIP1*/exp(*PDGFB*)))) > 1.182

**Table 3:** Performance of the selected meta-rule generated from the set of 70 genes on the validation set and result metrics.

	**Pathologically Node Positive**	**Pathologically Node Negative**
**Predicted Node Positive by GP using 70 genes**	6^§^	2
**Predicted Node Negative by GP using 70 genes**	4	19^†^

^§ ^True positive subjects.

^† ^True negative subjects.

Accuracy: 81%

Sensitivity: 60%

Specificity: 90%

Positive Predictive Value: 75%

Negative Predictive Value: 83%

**Table 4:** Probability of gene usage from the set of 70 genes due to random chance.

**Gene**	**Actual occurrence of gene (in 220 rules)**	**Binomial probability**	**p-value (one-sided)**
*KDR*	159	9.69E-130	<0.00001
*MAP2K6*	146	1.13E-110	<0.00001
*ICAM1*	121	4.10E-78	<0.00001
*ANXA5*	60	7.04E-20	<0.00001
*CDK8*	56	3.38E-17	<0.00001
*CDKN1A*	55	1.49E-16	<0.00001
*JUNB*	49	6.56E-13	<0.00001
*TGFBR2*	41	1.08E-08	<0.00001
*TNF*	24	1.11E-02	0.01490
*TNFAIP1*	23	1.76E-02	0.02599
*CCND3*	19	6.73E-02	0.16020
*PDGFRL*	18	8.23E-02	0.22749
*MAPK12*	15	1.04E-01	0.49264
*GAPDH*	15	1.04E-01	0.49264
*PTGS2*	12	7.05E-02	0.20295
*PDGFB*	11	5.26E-02	0.13246

Number of genes per rule = 5 (approximately)

Random occurrence of gene=Number of rules×Number of genes per ruleTotal number of genes=220×570=15.71
 MathType@MTEF@5@5@+=feaafiart1ev1aaatCvAUfKttLearuWrP9MDH5MBPbIqV92AaeXatLxBI9gBaebbnrfifHhDYfgasaacH8akY=wiFfYdH8Gipec8Eeeu0xXdbba9frFj0=OqFfea0dXdd9vqai=hGuQ8kuc9pgc9s8qqaq=dirpe0xb9q8qiLsFr0=vr0=vr0dc8meaabaqaciaacaGaaeqabaqabeGadaaakeaafaqadeGabaaabaGaeeOuaiLaeeyyaeMaeeOBa4MaeeizaqMaee4Ba8MaeeyBa0MaeeiiaaIaee4Ba8Maee4yamMaee4yamMaeeyDauNaeeOCaiNaeeOCaiNaeeyzauMaeeOBa4Maee4yamMaeeyzauMaeeiiaaIaee4Ba8MaeeOzayMaeeiiaaIaee4zaCMaeeyzauMaeeOBa4MaeeyzauMaeyypa0JaeeOta4KaeeyDauNaeeyBa0MaeeOyaiMaeeyzauMaeeOCaiNaeeiiaaIaee4Ba8MaeeOzayMaeeiiaaIaeeOCaiNaeeyDauNaeeiBaWMaeeyzauMaee4CamNaey41aq7aaSaaaeaacqqGobGtcqqG1bqDcqqGTbqBcqqGIbGycqqGLbqzcqqGYbGCcqqGGaaicqqGVbWBcqqGMbGzcqqGGaaicqqGNbWzcqqGLbqzcqqGUbGBcqqGLbqzcqqGZbWCcqqGGaaicqqGWbaCcqqGLbqzcqqGYbGCcqqGGaaicqqGYbGCcqqG1bqDcqqGSbaBcqqGLbqzaeaacqqGubavcqqGVbWBcqqG0baDcqqGHbqycqqGSbaBcqqGGaaicqqGUbGBcqqG1bqDcqqGTbqBcqqGIbGycqqGLbqzcqqGYbGCcqqGGaaicqqGVbWBcqqGMbGzcqqGGaaicqqGNbWzcqqGLbqzcqqGUbGBcqqGLbqzcqqGZbWCaaaabaGaeyypa0JaeGOmaiJaeGOmaiJaeGimaaJaey41aq7aaSaaaeaacqaI1aqnaeaacqaI3aWncqaIWaamaaGaeyypa0JaeGymaeJaeGynauJaeiOla4IaeG4naCJaeGymaedaaaaa@AAAE@

Arranged in decreasing order of their frequencies of occurrence in 220 rules, the genes show a general trend towards increasing probability of being selected in a rule by random chance. The postulated frequency of occurrence of each gene if all have equal probabilities of being selected is 15.71. *PTGS2 *and *PDGFB *have smaller probabilities than the two genes preceding them because they were actually used less frequently than random chance would suggest.

**Table 5:** Performance of the meta-rule generated using the three most frequently used genes, viz.* KDR*, *MAP2K6 *and *ICAM1*, on the validation set and result metrics.

	**Pathologically Node Positive**	**Pathologically Node Negative**
**Predicted Node Positive by GP using 3 genes**	7^§^	3
**Predicted Node Negative by GP using 3 genes**	3	18^†^

^§ ^True positive subjects

^† ^True negative subjects

Accuracy: 81%

Sensitivity: 70%

Specificity:86%

Positive Predictive Value: 70%

Negative Predictive Value: 86%

**Table 6:** Advantages of genetic programming.

**Method**	**Human Readability**	**Automatic Selection of Variables**	**Automatic Integration of Data Types**	**Non-Linear Relationships**
Statistical Analysis	Yes	Limited	No	Limited
Cluster Analysis	Yes	No	No	No
Support Vector Machine	No	No	No	Yes
Neural Networks	No	No	No	Yes
**Genetic Programming**	Yes	Yes	Yes	Yes
