# Supplementary material for: The use of genetic programming in the analysis of quantitative gene expression profiles for identification of nodal status in bladder cancer
Source: BMC Cancer. 2006 Jun 16;6:159. doi: 10.1186/1471-2407-6-159 (PMC1550424; doi:10.1186/1471-2407-6-159)
Supplement: Additional file 1 — A comprehensive list of the genes investigated using StaRT-PCR™ including their GenBank accession, GeneID and UniGene cluster numbers. [file 1471-2407-6-159-S1.pdf]

Additional File 1

Gene	Gene Aliases	Name	GenBank Accession	GeneID	UniGene cluster
Apoptosis					
ANXA5	ANX5; ENX2; PP4	annexin A5	NM_001154.2	308	Hs.480653
BAD	BBC2; BCL2L8	BCL2-antagonist of cell death	NM_032989.1 / NM_004322.2	572	Hs.370254
BCL2	Bcl-2	B-cell CLL/lymphoma 2	NM_000633.2	596	Hs.592350
BCL2L1	BCLX; BCL2L; Bcl-X; bcl-xL; bcl-xS; BCL-XL/S; DKFZp781P2092	BCL2-like 1	NM_138578.1	598	Hs.516966
CYP1A2	CP12; P3-450; P450(PA)	cytochrome P450, family 1, subfamily A, polypeptide 2	NM_000761.3	1544	Hs.1361
DAP	MGC99796	death-associated protein	NM_004394.1	1611	Hs.75189
PTGS2	COX2; COX-2; PHS-2; PGG/HS; PGHS-2; hCox-2	prostaglandin-endoperoxide synthase 2 (prostaglandin G/H synthase and cyclooxygenase)	NM_000963.1	5743	Hs.196384
TGFBR2	AAT3; FAA3; MFS2; RIIC; TAAD2; HNPCC6; TGFR-2; TGFbeta-RII	transforming growth factor, beta receptor II (70/80kDa)	NM_003242.4	7048	Hs.604277
TGIF	HPE4; MGC5066; MGC39747	TGFB-induced factor (TALE family homeobox)	NM_003244.2	7050	Hs.373550
TNF	DIF; TNFA; TNFSF2; TNF-alpha	tumor necrosis factor (TNF superfamily, member 2)	NM_000594.2	7124	Hs.241570
TNFAIP1	B61; EDP1	tumor necrosis factor, alpha-induced protein 1 (endothelial)	NM_021137.3	7126	Hs.76090
TNFRSF1A	CD120a; FPF; MGC19588; TBP1; TNF-R; TNF-R-I; TNF-R55; TNFAR; TNFR1; TNFR55; TNFR60; p55; p55-R; p60	tumor necrosis factor receptor superfamily, member 1A	NM_001065.2	7132	Hs.279594
TNFSF10	TL2; APO2L; CD253; TRAIL; Apo-2L	tumor necrosis factor (ligand) superfamily, member 10	NM_003810.2	8743	Hs.478275
TRAF4	CART1; MLN62; RNF83	TNF receptor-associated factor 4	NM_004295.2	9618	Hs.8375

Gene	Gene Aliases	Name	GenBank Accession	GeneID	UniGene cluster
Cell Cycle					
CCNA2	CCN1; CCNA	cyclin A2	NM_001237.2	890	Hs.58974
CCND3		cyclin D3	NM_001760.2	896	Hs.534307
CCNE1	CCNE	cyclin E1	NM_001238.1 / NM_057182.1	898	Hs.244723
CCNG1	CCNG	cyclin G1	NM_004060.3 / NM_199246.1	900	Hs.79101
CDC2	CDK1; MGC111195; DKFZp686L20222	cell division cycle 2, G1 to S and G2 to M	NM_001786.2	983	Hs.334562
CDC25C	CDC25	cell division cycle 25C	NM_001790.2 / NM_022809.1	995	Hs.656
CDK7	CAK1; STK1; CDKN7; p39MO15	cyclin-dependent kinase 7 (MO15 homolog, Xenopus laevis, cdk-activating kinase)	NM_001799.2	1022	Hs.184298
CDK8	K35; MGC126074; MGC126075	cyclin-dependent kinase 8	NM_001260.1	1024	Hs.382306
PCNA	MGC8367	proliferating cell nuclear antigen	NM_002592.2/ NM_182649.1	5111	Hs.147433

Gene	Gene Aliases	Name	GenBank Accession	GeneID	UniGene cluster
Gene Regulation					
FOS	c-fos	v-fos FBJ murine osteosarcoma viral oncogene homolog	NM_005252.2	2353	Hs.25647
FOSL1	FRA1; fra-1	FOS-like antigen 1	NM_005438.2	8061	Hs.283565
HSF1	HSTF1	heat shock transcription factor 1	NM_005526.1	3297	Hs.530227
MAP3K14	HS; NIK; HSNIK; FTDCR1B	mitogen-activated protein kinase kinase kinase 14	NM_003954.1	9020	Hs.404183
SP1		Sp1 transcription factor	NM_138473.2	6667	Hs.620754
JUN	AP1	v-jun sarcoma virus 17 oncogene homolog (avian)	NM_002228.3	3725	Hs.525704
JUNB		jun B proto-oncogene	NM_002229.2	3726	Hs.25292
MAX	orf1; MGC10775; MGC11225; MGC18164; MGC34679; MGC36767	MYC associated factor X	NM_145113.1	4149	Hs.285354
MYC	c-Myc	v-myc myelocytomatosis viral oncogene homolog (avian)	NM_002467.3	4609	Hs.202453
NFKB1	KBF1; EBP-1; MGC54151; NFKB-p50; NFKB-p105; NF-kappa-B; DKFZp686C01211	nuclear factor of kappa light polypeptide gene enhancer in B-cells 1 (p105)	NM_003998.2	4790	Hs.431926

Gene	Gene Aliases	Name	GenBank Accession	GeneID	UniGene cluster
Apoptosis + Cell Cycle					
RB	RB; OSRC	retinoblastoma 1 (including osteosarcoma)	NM_000321.1	5925	Hs.408528
RBL2	Rb2; P130; FLJ26459	retinoblastoma-like 2 (p130)	NM_005611.2	5934	Hs.513609
CDKN1A	P21; CIP1; SDI1; WAF1; CAP20; CDKN1; MDA-6; p21CIP1	cyclin-dependent kinase inhibitor 1A (p21, Cip1)	NM_000389.2 / NM_078467.1	1026	Hs.370771
CDKN1B	KIP1; CDKN4; P27KIP1	cyclin-dependent kinase inhibitor 1B (p27, Kip1)	NM_004064.2	1027	Hs.238990
CDKN2A	ARF; MLM; p14; p16; p19; CMM2; INK4; MTS1; TP16; CDK4I; CDKN2; INK4a; p14ARF; p16INK4; p16INK4a	cyclin-dependent kinase inhibitor 2A (melanoma, p16, inhibits CDK4)	NM_000077.3	1029	Hs.512599
CDKN2C	p18; INK4C; p18-INK4C	cyclin-dependent kinase inhibitor 2C (p18, inhibits CDK4)	NM_001262.2 / NM_078626.1	1031	Hs.525324
GAPDH	G3PD; GAPD; MGC88685	glyceraldehyde-3-phosphate dehydrogenase	NM_002046.2	2597	Hs.592355
MXD1	MAD; MAD1; MGC104659	MAX dimerization protein 1	NM_002357.2	4084	Hs.468908
TP53	p53; LFS1; TRP53	tumor protein p53 (Li-Fraumeni syndrome)	NM_000546.2	7157	Hs.408312

Gene	Gene Aliases	Name	GenBank Accession	GeneID	UniGene cluster
Apoptosis + Cell Cycle + Gene Regulation					
E2F1	RBP3; E2F-1; RBBP3	E2F transcription factor 1	NM_005225.1	1869	Hs.516986
E2F2	E2F-2	E2F transcription factor 2	NM_004091.2	1870	Hs.194333
E2F4	E2F-4	E2F transcription factor 4, p107/p130-binding	NM_001950.3	1874	Hs.108371
E2F5	E2F-5	E2F transcription factor 5, p130-binding	NM_001951.2	1875	Hs.445758
Anti-oxidation					
GSTM3	GST5; GSTB; GTM3; GSTM3-3; MGC3310; MGC3704	glutathione S-transferase M3 (brain)	NM_00849.3	2947	Hs.2006
GSTP1	PI; DFN7; GST3; FAEES3	glutathione S-transferase pi	NM_000852.2	2950	Hs.523836
GSTT1		glutathione S-transferase theta 1	NM_000853.1	2952	Hs.268573
SOD1	ALS; SOD; ALS1; IPOA; homodimer	superoxide dismutase 1, soluble (amyotrophic lateral sclerosis 1 (adult))	NM_000454.4	6647	Hs.443914

Gene	Gene Aliases	Name	GenBank Accession	GeneID	UniGene cluster
Cell Growth Regulation					
IGF1	IGFI	insulin-like growth factor 1 (somatomedin C)	NM_000618.2	3479	Hs.160562
IGF2R	MPRI; CD222; CIMPR; M6P-R	insulin-like growth factor 2 receptor	NM_000876.1	3482	Hs.487062
PDGFB	SIS; SSV; PDGF2; c-sis; FLJ12858	platelet-derived growth factor beta polypeptide (simian sarcoma viral (v-sis) oncogene homolog)	NM_002608.1 / NM_033016.1	5155	Hs.1976
PDGFRL	PDGRL; PRLTS	platelet-derived growth factor receptor-like	NM_006207.1	5157	Hs.458573
Signal Transduction					
ERBB2	NEU; NGL; HER2; TKR1; HER-2; c-erb B2; HER-2/neu	v-erb-b2 erythroblastic leukemia viral oncogene homolog 2, neuro/glioblastoma derived oncogene homolog (avian)	NM_004448.2 / NM_001005862.1	2064	Hs.446352
MAPK8	JNK; JNK1; PRKM8; SAPK1; JNK1A2; JNK21B1/2	mitogen-activated protein kinase 8	NM_139049.1 / NM_002750.2	5599	Hs.138211
MAPK9	JNK2; JNK2A; JNK2B; PRKM9; JNK-55; JNK2BETA; p54aSAPK; JNK2ALPHA	mitogen-activated protein kinase 9	NM_139069.1 / NM_139070.1	5601	Hs.484371
MAPK12	ERK3; ERK6; SAPK3; PRKM12; SAPK-3; P38GAMMA	mitogen-activated protein kinase 12	NM_002969.3	6300	Hs.432642
MAP2K6	MEK6; MKK6; MAPKK6; PRKMK6; SAPKK3	mitogen-activated protein kinase kinase 6	NM_002758.2 / NM_ 031988.1	5608	Hs.463978
STAT3	APRF; FLJ20882; MGC16063	signal transducer and activator of transcription 3 (acute-phase response factor)	NM_139276.2	6774	Hs.463059
LYN	JTK8; FLJ26625	v-yes-1 Yamaguchi sarcoma viral related oncogene homolog	NM_002350.1	4067	Hs.491767

Gene	Gene Aliases	Name	GenBank Accession	GeneID	UniGene cluster
Angiogenesis					
FGF5	HBGF-5; Smag-82	fibroblast growth factor 5	NM_004464.3 / NM_033143.2	2250	Hs.37055
FGFR4	TKF; JTK2; CD334; MGC20292	fibroblast growth factor receptor 4	NM_002011.3	2264	Hs.165950
VEGF	VPF; VEGFA; MGC70609	vascular endothelial growth factor	NM_003376.4	7422	Hs.73793
KDR	FLK1; CD309; VEGFR; VEGFR2	kinase insert domain receptor (a type III receptor tyrosine kinase)	NM_002253.1	3791	Hs.479756
Invasion					
BMP6	VGR; VGR1	bone morphogenetic protein 6	NM_001718.2	654	Hs.285671
CDH3	CDHP; HJMD; PCAD	cadherin 3, type 1, P-cadherin (placental)	NM_001793.3	1001	Hs.554598
ICAM1	BB2; CD54; P3.58	intercellular adhesion molecule 1 (CD54), human rhinovirus receptor	NM_000201.1	3383	Hs.515126
MMP16	MMP-X2; MT-MMP2; MT-MMP3; MT3-MMP	matrix metallopeptidase 16 (membrane-inserted)	NM_005941.2 / NM_022564.1	4325	Hs.546267
TIMP2	CSC-21K	TIMP metallopeptidase inhibitor 2	NM_003255.4	7077	Hs.104839
Reference Gene					
ACTB	PS1TP5BP1	actin, beta	NM_001101.2	60	Hs.520640

Detailed list of genes investigated by StaRT-PCR™

The entire study analyzed 70 candidate markers within eight partially overlapping pathways involved in carcinogenesis. The above tables list comprehensive information on each gene based on the records maintained by the United States National Library of Medicine. GenBank accession and GeneID numbers are provided through Entrez Gene and UniGene cluster numbers are provided through UniGene. All hyperlinks on this document are current as of June 08, 2006.
