# Supplementary material for: The use of genetic programming in the analysis of quantitative gene expression profiles for identification of nodal status in bladder cancer
Source: BMC Cancer. 2006 Jun 16;6:159. doi: 10.1186/1471-2407-6-159 (PMC1550424; doi:10.1186/1471-2407-6-159)
Supplement: Additional file 2 — The box plots for the gene expression values for KDR, MAP2K6 and ICAM1 that are differentially expressed in the primary tumor tissues of the node positive and the node negative classes. [file 1471-2407-6-159-S2.pdf]

The use of genetic programming in the analysis of quantitative gene expression profiles for identification of nodal status in bladder cancer

Mitra AP, Almal AA, George B, Fry DW, Lenehan PF, Pagliarulo V, Cote RJ, Datar RH, Worzel WP

Additional File 2

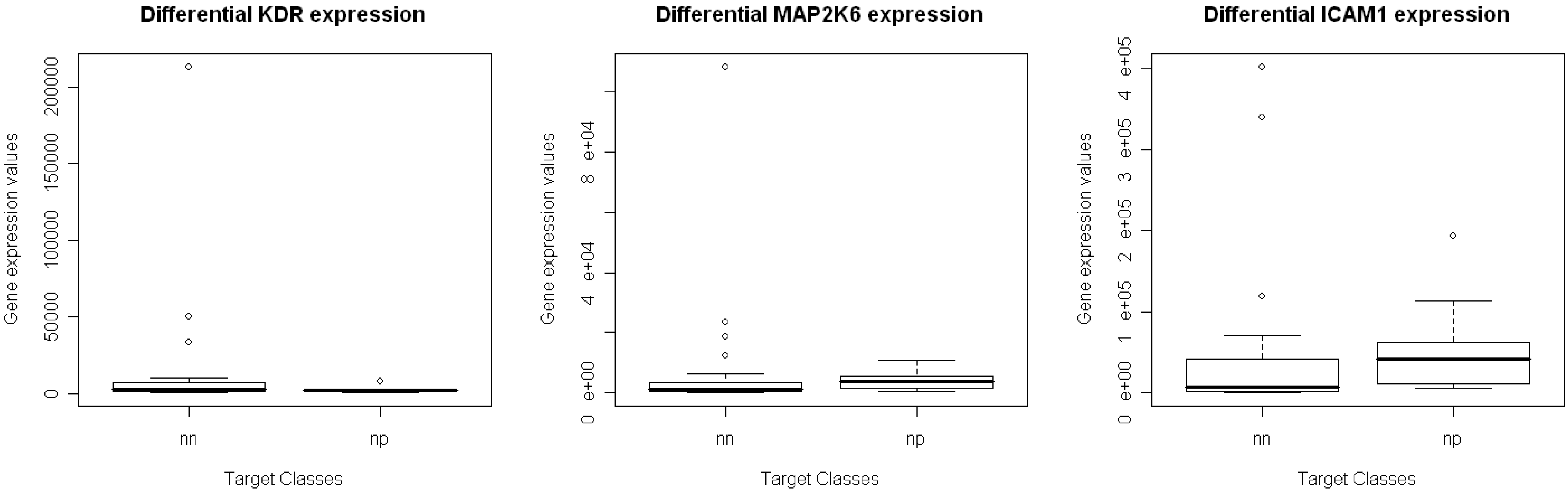

Box plots for gene expression values for *KDR*, *MAP2K6* and *ICAM1*

The box plots show that the genes *KDR*, *MAP2K6* and *ICAM1* are differentially expressed in the node positive and the node negative cases. However, the figure also indicates that these genes, either independently or in a linear combination, cannot be used to discriminate between the node positive and the node negative classes. Relative comparisons of the gene expression values, however, can be used as indicators of nodal status.
