# Supplementary material for: The use of genetic programming in the analysis of quantitative gene expression profiles for identification of nodal status in bladder cancer
Source: BMC Cancer. 2006 Jun 16;6:159. doi: 10.1186/1471-2407-6-159 (PMC1550424; doi:10.1186/1471-2407-6-159)
Supplement: Additional file 3 — The classifier rules that form the meta-rule generated for node positive patients using the three most frequently used genes, viz. KDR, MAP2K6 and ICAM1. [file 1471-2407-6-159-S3.pdf]

Additional File 3

Rule number	Classifier Rule
1	(*MAP2K6* / *KDR*) x (1.0 - (*MAP2K6* / *ICAM1*)) > .71
2	*MAP2K6* x ((*ICAM1* - *KDR*) / (*ICAM1* x *KDR*)) > .705
3	*MAP2K6* x ((*ICAM1* - *KDR*) / (*ICAM1* x *KDR*)) > .705
4	(*MAP2K6* / *KDR*) - exp(*KDR*) - (*MAP2K6* / *ICAM1*) > -.294
5	(*MAP2K6* / *KDR*) x (1.0 - (*MAP2K6* / *ICAM1*)) > .71
6	*ICAM1* / (*MAP2K6* - *KDR* x exp(*ICAM1*)) > 4.092
7	(*MAP2K6* / *KDR*) x (1.0 - (*MAP2K6* / *ICAM1*)) > .71
8	(*MAP2K6* / *KDR*) - (*MAP2K6* / (*ICAM1* + exp(*ICAM1*))) > .705
9	*MAP2K6* x ((*ICAM1* - *KDR*) / (*ICAM1* x *KDR*)) > .69
10	(*MAP2K6* / *KDR*) - (*MAP2K6* / (*ICAM1* x exp(*KDR*))) > .705
11	(*MAP2K6* / *KDR*) - (*MAP2K6* / (*ICAM1* + exp(*ICAM1*))) > .886

Meta-rule for node positive patients using the three most frequently used genes, viz. *KDR*, *MAP2K6* and *ICAM1*.
Note that rules 1, 5 and 7 feature the same rule.
