# Supplementary material for: The use of genetic programming in the analysis of quantitative gene expression profiles for identification of nodal status in bladder cancer
Source: BMC Cancer. 2006 Jun 16;6:159. doi: 10.1186/1471-2407-6-159 (PMC1550424; doi:10.1186/1471-2407-6-159)
Supplement: Additional file 4 — The performance metrics of the first classifier rule from the meta-rule generated for node positive patients using the three most frequently used genes, viz. KDR, MAP2K6 and ICAM1, on the validation set. [file 1471-2407-6-159-S4.pdf]

Additional File 4

	Pathologically Node Positive	Pathologically Node Negative
**Predicted Node Positive by GP using a single rule**	7[§]	0
**Predicted Node Negative by GP using a single rule**	3	21[†]

[§] True positive subjects
[†] True negative subjects

Accuracy: 90%
Sensitivity: 70%
Specificity: 100%
Positive Predictive Value: 100%
Negative Predictive Value: 88%

Performance of the rule "(*MAP2K6* / *KDR*) x (1.0 - (*MAP2K6* / *ICAM1*)) > .71" selected from the meta-rule generated using the three most frequently used genes, viz. *KDR*, *MAP2K6* and *ICAM1*, on the validation set and result metrics.
