# Supplementary material for: The use of genetic programming in the analysis of quantitative gene expression profiles for identification of nodal status in bladder cancer
Source: BMC Cancer. 2006 Jun 16;6:159. doi: 10.1186/1471-2407-6-159 (PMC1550424; doi:10.1186/1471-2407-6-159)
Supplement: Additional file 5 — The stage-wise and case-wise performance of the meta-rules generated using 70 genes and the three most frequently used genes, viz. KDR, MAP2K6 and ICAM1, on the validation set. This file also shows the stage- and case-wise performance of the single rule "(MAP2K6/KDR) × (1.0 - (MAP2K6/ICAM1)) > .71" selected from the meta-rule generated using the three most frequently used genes on the validation set. [file 1471-2407-6-159-S5.pdf]

Additional File 5

Pathological stage		Number of subjects		Number of validation set subjects correctly predicted by GP		
**Lymph node**	**Tumor**	**Training set**	**Validation set**	**Meta-rule using 70 genes**	**Meta-rule using 3 genes§**	**Using single rule†**
Node positive	pTa	0	0	n/a	n/a	n/a
	pT1	2	1	1	1	1
	pT2	0	0	n/a	n/a	n/a
	pT3	7	7	3	4	4
	pT4	2	2	2	2	2
Node negative	Normal controls	3	2	2	2	2
	pTa	3	7	7	7	7
	pT1	6	4	4	4	4
	pT2	4	4	4	2	4
	pT3	5	3	2	3	3
	pT4	2	1	0	0	1

Table 5-1: Stage-wise performance of the GP runs on the validation set.

Subject	Pathological stage		Prediction of nodal stage of validation set subjects by GP		
	Tumor	Lymph node	Meta-rule using 70 genes	Meta-rule using 3 genes§	Using single rule†
VS1	pTa	NN	✓	✓	✓
VS2	pTa	NN	✓	✓	✓
VS3	pTa	NN	✓	✓	✓
VS4	Normal	NN	✓	✓	✓
VS5	pTa	NN	✓	✓	✓
VS6	pTa	NN	✓	✓	✓
VS7	pTa	NN	✓	✓	✓
VS8	pT2	NN	✓	✗	✓
VS9	pT2	NN	✓	✓	✓
VS10	pT1	NN	✓	✓	✓
VS11	pT1	NN	✓	✓	✓
VS12	pT1	NP	✓	✓	✓
VS13	Normal	NN	✓	✓	✓
VS14	pT1	NN	✓	✓	✓
VS15	pT1	NN	✓	✓	✓
VS16	pT3	NP	✗	✓	✓
VS17	pT4	NN	✗	✗	✓
VS18	pT4	NP	✓	✓	✓
VS19	pT3	NN	✓	✓	✓
VS20	pT3	NN	✓	✓	✓
VS21	pT3	NP	✓	✓	✓
VS22	pT3	NP	✗	✗	✗
VS23	pT3	NN	✗	✓	✓
VS24	pT2	NN	✓	✓	✓
VS25	pT3	NP	✗	✗	✗
VS26	pT3	NP	✓	✓	✓
VS27	pT3	NP	✓	✓	✓
VS28	pTa	NN	✓	✓	✓
VS29	pT2	NN	✓	✗	✓
VS30	pT3	NP	✗	✗	✗
VS31	pT4	NP	✓	✓	✓

Table 5-2: Case-wise performance of the GP runs on the validation set.

Key:

§ Meta-rule generated using the three most frequently used genes, viz. *KDR*, *MAP2K6* and *ICAM1*

† Rule “(*MAP2K6* / *KDR*) x (1.0 - (*MAP2K6* / *ICAM1*)) > .71” selected from the meta-rule generated using the three most frequently used genes, viz. *KDR*, *MAP2K6* and *ICAM1*

✓ Correct prediction of pathological nodal status
✗ Incorrect prediction of pathological nodal status
